# Dendrimers for siRNA Delivery

**DOI:** 10.3390/ph6020161

**Published:** 2013-02-04

**Authors:** Swati Biswas, Vladimir P. Torchilin

**Affiliations:** Center for Pharmaceutical Biotechnology and Nanomedicine, 360 Huntington Ave, 140 The Fenway, Northeastern University, Boston, MA 02115, USA; E-Mail: sbiswaswsu@gmail.com

**Keywords:** dendrimer, siRNA, delivery

## Abstract

Since the discovery of the “starburst polymer”, later renamed as dendrimer, this class of polymers has gained considerable attention for numerous biomedical applications, due mainly to the unique characteristics of this macromolecule, including its monodispersity, uniformity, and the presence of numerous functionalizable terminal groups. In recent years, dendrimers have been studied extensively for their potential application as carriers for nucleic acid therapeutics, which utilize the cationic charge of the dendrimers for effective dendrimer-nucleic acid condensation. siRNA is considered a promising, versatile tool among various RNAi-based therapeutics, which can effectively regulate gene expression if delivered successfully inside the cells. This review reports on the advancements in the development of dendrimers as siRNA carriers.

## 1. Introduction

Interference of the cellular pathway of protein synthesis by a RNA interference mechanism has been considered the most powerful approach by which over-expression of aberrant proteins can be successfully minimized [1,2,3]. RNA interference (RNAi) is the mechanism adopted by most eukaryotic cells, where by small double-stranded RNA (dsRNA) molecules control gene expression by potent degradation of its complementary messenger RNA (mRNA) sequence, commonly referred to in plants as post-transcriptional gene silencing (PTGS) [4,5]. While transcriptional gene silencing involves mutations to specific regions of the genes that results in the production of proteins with non-functional domains, PTGS involves repression of gene expression through specific mRNA degradation [1,6].

Since their discovery less than a decade ago, RNAi-based therapeutics have found their way into clinical trials [7,8,9]. Gene silencing at the post-transcriptional level can be achieved by three basic mechanisms, including the use of antisense oligo-deoxyribonucleic acids (ODN), ribozymes and dsRNA [10]. RNAi by double-stranded small interfering RNA (siRNA) is considered the most powerful technique since-specific siRNA complementary to the target mRNA elicits potent target-specific knockdown of any mRNA [10,11]. Since the discovery of their ability to interfere with a disease-causing aberrant protein at the level of gene expression, siRNAs have garnered considerable attention as potential therapeutic agents for the treatment of cancers and other related diseases [2,12]. However, the successful use of this molecule faces challenges for *in vivo* delivery [13,14,15]. Low resistance against enzymatic degradation, limited translocation across the cell membrane and a substantial liver clearance limit the applications [16]. Therefore, development of an efficient delivery system requires protection of siRNA from degradation, limitation of rapid renal clearance, and promotion of targeted intracellular delivery for effective knockdown of protein synthesis with minimal side-effects.

For decades, cationic polymers, lipids and polyamino acids, have been used extensively as carriers for nucleic acid therapeutics [17,18,19,20,21,22,23,24]. The cationic charge of the carriers allows electrostatic interaction with the anionic nucleic acid molecules that leads to effective condensation. These nanosized siRNA-polymer complexes can protect nucleic acids from non-specific interactions and enzymatic degradation in the systemic circulation. The cationic polymers utilized for nucleic acid delivery applications include low-and high-molecular weight poly(ethyleneimines), cationic poly-saccharides, such as chitosan, dendrimers, polypeptides such as poly-L-lysines, polyarginines and various cationic lipids [25,26,27,28,29,30,31,32,33].

Dendrimers are a relatively new class of cationic polymers, which have been studied extensively as potential nucleic acid carriers [34,35,36,37,38]. Since the synthesis of a “cascade molecule” in 1978, and later, a starburst polymer in 1985, such hyperbranched macromolecules with a defined core and repetitively attached exterior units have been synthesized with a variety of chemical structures. Dendrimers are highly symmetric, spherical, hyperbranched macromolecules having a tunable structure, molecular size, and surface charge. Their unique structural features including chemical homogeneity, the possibility of increasing the generation by repeated attachment of chemical groups, and a high density of functional groups on the surface for numerous ligand attachments make dendrimers an excellent polymer candidate for numerous biomedical applications.

Polycationic forms such as poly(amidoamine) (PAMAM) and poly(propyleneimine) (PPI) dendrimers have been studied extensively as drug/gene carriers [36,37,38,39,40]. However, the potential of dendrimers as a siRNA delivery vector remains relatively unexplored. This paper reviews the major obstacles for translating RNAi from a genomic tool into clinical practice and the recent progress made in developing nano-scale siRNA delivery systems utilizing dendrimers as the cationic polymer.

## 2. siRNA-Mediated Gene Silencing

Small interfering RNA (siRNA) with 19-21 base pairs has been recognized as a therapeutic agent for effectively silencing a disease-related gene on a post-transcriptional level [3,4,9]. siRNA therapeutics interfere with the RNAi pathway by inhibiting the translation of a complementary mRNA. In cellular systems, RNAi can be triggered by two basic pathways in which the ultimate effector molecule is a small 21–23 nucleotide antisense RNA. One approach utilizes a relatively long dsRNA which is processed by the cellular Dicer enzyme into short 21–23 nucleotide dsRNA, referred to as siRNA. Synthetically prepared exogenous siRNA, complementary to the mRNA of a specific disease-causing protein can be transferred to the cells. The other cellular approach uses short hairpin RNAs (shRNAs) that are transported from the nucleus to the cytoplasm via the microRNA (miRNA) machinery. In the nucleus, transcription of a long primary miRNA by RNA polymerase II produces a stem-loop structured miRNA of ~70 nucleotides which is termed precursor miRNA (pre-miRNA) [41]. dsRNA binding protein, Exportin-5 chaperones this pre-miRNA to the cytoplasm. Once in the cytoplasm, Dicer, an endoribonuclease in the RNAse III family, cleaves pre-mRNA to a mature miRNA duplex of 22 nucleotide (nt) length with 5'-phosphorylated ends and 2-nt 3' overhangs. A ribonucleoprotein complex, RNA-induced silencing complex (RISC) unwinds the RNA duplex and discards the sense strand [42]. The antisense strand of miRNA guides RISC to its target mRNA and binds partially with the mRNA transcript in a complementarity-dependent manner [43,44].

siRNA used for therapeutic application is a chemically synthesized RNA duplex, 19-23 nt in length. siRNA has a 2-nt 3' overhang, similar to endogenous miRNA that allows Dicer to recognize and further process them in the same way as miRNA. Generally, siRNA consists of two separate, annealed single strands of 21 nucleotides, where the terminal two 3'-nucleotides are unpaired (3' overhang). siRNA can also be in the form of a single stem-look, termed a short hairpin RNA (shRNA). Unlike miRNA, the antisense strand of siRNA is completely complementary to the mRNA target site. Typically, but not always, the antisense strand is complementary to the sense strand of the siRNA. Binding of siRNA strand-incorporated RISC to the target mRNA complementary to a single siRNA strand initiates the cleavage of the mRNA strand within the target site, which leads to translational repression followed by mRNA degradation. The siRNA strand can be recycled to bind with RISC for a repetitive action of mRNA degradation. Even though direct use of siRNA is simple and efficient for gene silencing, the effect is transient and so requires repeated administration for effectiveness. The DNA-based RNAi approach for gene therapy is stable and thus requires only a single treatment with shRNA genes.

## 3. Advantages of siRNA for Therapy

Generally, RNAi can be considered an effective strategy to combat disease progression over conventional therapeutics because of its ability to repress translation of any disease-causing protein via gene silencing. The Watson-Crick base pairing interactions of the RNA molecules with the target specifically discriminate between target and off-targets [7]. Among all the RNA molecules causing gene-specific silencing via the RNAi pathway including siRNA, shRNA and miRNA, the antisense strand of siRNA is completely complementary to the mRNA target and has higher target recognition and binding compared to other RNA molecules which are partially complementary to the target mRNA [7,45]. With our currently improved understanding of the intricate molecular mechanisms of many diseases and the regulation of disease-related biomarkers, it is possible to determine the molecular signature of each patient's specific disease to enable development of a tailored therapy with increased efficacy and safety profiles [9]. Complete understanding of the methodology for siRNA synthesis enables preparation of a siRNA that selectively turns off a targeted disease-causing gene. In general, antisense drugs can be designed for synthesis based on the target mRNA sequence and, theoretically, any gene can be silenced using an antisense strategy. Owing to the high potency and minimum off-target interaction of siRNA among all the antisense molecules, use of siRNA has been considered the most promising tool being applied to personalized medicine [46].

Gene silencing using RNAi technology has advantages over conventional therapeutic. The inhibitory effect of conventional drugs is achieved mainly by blocking a protein’s function by binding to the protein's active site, where the receptor-ligand interaction takes place leading to downstream activation of signaling pathways. However, the small molecule-mediated drug action may not be achieved if the disease-related protein has a conformation that is not accessible to small molecules, which is then referred to as a “non-druggable” target [9,47]. Design and synthesis of small molecule ligands for inhibition of a target protein is challenging and there is always a possibility for discovery of novel ligands, lead molecules and optimization to achieve higher inhibition than an existing ligand. On the other hand, RNAi technology allows blocking of the gene expression of the target protein rather than blocking the activity of the target protein [48,49]. Synthesis of RNAi molecules complementary to any gene is relatively easy and can be applied using well demonstrated strategies compared to widely varied small molecule synthesis.

## 4. Challenges

Therapeutic application of all RNAi molecules, including siRNA, faces challenges related to their delivery. The naked siRNA is highly unstable and rapidly degraded by serum nucleases when administered systemically [7,50]. Chemical modification in the siRNA molecule may be used to overcome this limitation [50]. However, modification should be tolerated by the RNAi machinery. Since the 2'-OH group of the ribose in the RNA molecule is not essential for siRNA recognition by RNAi machinery, diverse modifications at the 2'-position in both the strands have been done extensively, and are referred to as 2'-modifications [51]. Examples of such modifications include an ether linkage as a 2'-methylnucleoside (2'-O-Me) and replacement of the -OH with its bioisostere, F, as in 2-deoxyfluridine (2'-F) [51]. Another modification of the ribose at 2'-position involves introducing a methylene group between the 2'- and 4'-positions by an ether linkage (-O-CH_2_-O-), which is termed a locked nucleic acid (LNA) modification [52]. The modifications done on the phosphate backbone at the 3'-end provides another effective approach to prevent siRNA degradation. Phosphothionate, boranophosphate, phosphoroamidate and methylphosphonate modifications are made between two riboses [50,53,54]. These modifications of siRNA enhance the stability in serum and thermal stability without compromising the efficiency of RNAi [53,54,55]. Chemical modifications of siRNA by attachment of bulky groups at the 2'-position adversely affect interaction with Dicer and loading into RISC [50,56].

Apart from the recognition by nucleases in the systemic circulation, naked, unmodified RNAi molecules undergo rapid renal clearance. Molecules of formula weight less than 50 KDa (~10 nm) appear in the glomerular filtrate of the kidney and are excreted [57]. Systemically administered siRNA accumulates preferentially in the kidney at a 40-fold higher concentration than in other organs and is excreted in the urine within an hour [13,58]. Naked siRNA also induces an immune response and activates circulating mononuclear phagocytosis as a defense mechanism against viral infection [59]. Stimulation of an immune response by siRNA leads to the production of pro-inflammatory cytokines including IL-6, TNF-α and triggers activation of the type I interferon pathway [60]. siRNA exhibits off-target gene silencing effects by interfering with other endogenous miRNA pathways. Different siRNA sequences against the same gene can generate similar gene silencing signatures. Off-target gene silencing occurs when other mRNA transcripts partially hybridize with the administered siRNA [61].

Application of siRNA therapeutics faces major challenge for delivery to the desired site of action [7]. Nanoparticle-mediated siRNA delivery offers advantages and has the potential for safe and effective delivery of siRNA with improvement of immune tolerance, pharmacokinetics and biodistribution profile [62]. Complexation of negatively charged siRNA with cationic polymers or nanoparticles is the basis for vector-mediated siRNA delivery. However, there is a distinct difference between the vector-mediated delivery of DNA and siRNA molecules. Due to the smaller size of siRNA compared to therapeutic plasmid-sized DNA, this molecule interacts less efficiently with cationic polymers [63]. siRNA is also less flexible compared to plasmid DNA, as indicated by its persistence length [64]. DNA behaves as a rigid rod if its length does not exceed 50 nm [65]. A double-stranded RNA molecule has a length of 70 nm, which makes it less flexible compared to plasmid DNA [64]. Generally, 260 base pairs is the minimum requirement for optimum flexibility for DNA [66,67]. Therefore, unlike plasmid DNA, an approximately 21–23 base-paired rigid rod-structured therapeutic siRNA does not compact efficiently when complexed with a vector and leads to incomplete encapsulation and the formation of undesirably large complexes [68,69]. Consequently, the usual cationic polymers proven effective for gene delivery are not necessarily optimal as siRNA delivery vectors [70].

## 5. Polymeric siRNA Delivery Systems

Carriers for siRNA are divided broadly into two categories: viral and non-viral. Non-viral carriers typically involve complexation of siRNA with positively charged vectors such as cationic polymers, cationic cell penetrating peptides, dendrimers and cationic lipids [15,19,21,22,23,24,25,26,27,28,29,30,31,32,33,37,71,72,73,74]. Non-viral vectors are preferred over viral vectors due to potential toxicities and immune reactions associated with viral vectors [75,76].

Various cationic polymers have been utilized to effectively deliver siRNA [20,22,75,77]. Cationic polymers or nanoparticles interact with negatively charged siRNA molecules via electrostatic interaction under physiological condition, stabilize the siRNA and enhance its intracellular delivery. Cationic polymers not only protect siRNA from enzymatic degradation, they also facilitate endosomal escape by a “proton sponge” effect [27,28] .The nanoparticles formed by polymer-siRNA complexes are termed polyplexes, with size ranging from 100 to 400 nm. Cationic polymers for siRNA delivery include biocompatible natural polymers such as chitosan, cyclodextrin, atelocollagen, protamine as well as synthetic cationic polymers such as polyethyleneimine (PEI), poly(L-lysine) (PLL), poly-D,L-lactide-co-glycolide (PLGA), poly(alkylcyanoacrylate), chitosan, gelatin and dendrimers [19,20,28,77,78,79,80]. Among all the polymers utilized for siRNA delivery, PEI has been used extensively [14,72,81,82,83,84,85]. The following discussion focuses on utilization of dendrimers, a unique, hyperbranched polymeric system for potential applications in siRNA delivery.

## 6. Dendrimers

**Figure 1:**
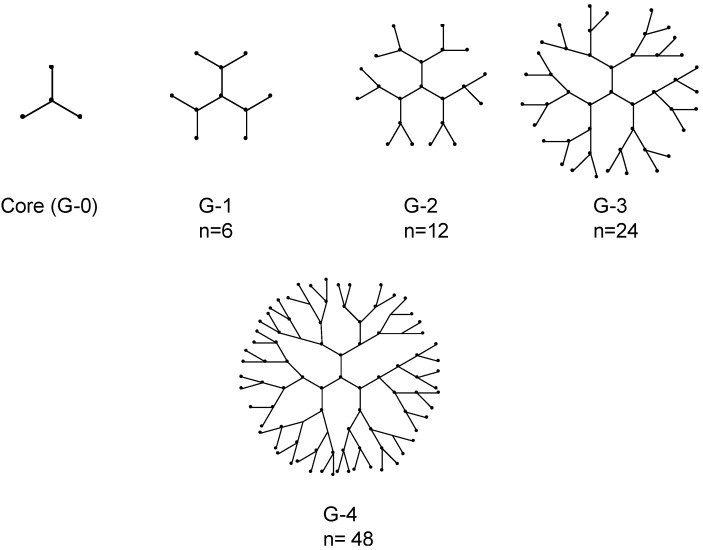
Representation of dendrimers of generations 1–4. The n denotes number of terminal functional groups.

Dendrimers have attracted a great deal of interest in areas ranging from drug and nucleic acid delivery applications to processing, diagnostics and nanoengineering due to their uniform, well-defined, three dimensional structures [34,36,37,40,86]. The name "dendrimer" originated from the Greek work “Dendron” meaning tree and “meros” meaning part, which depicts a structure that consists of a central core molecule that acts as a root, from which a number of highly branched, tree-like arms originates in a symmetrical manner (Figure 1).

Dendrimers differ from classical monomers or oligomers by their defined architecture, high branching from the center/core of the molecule and terminal functional group density. Key properties such as molecular symmetry and a high ratio of multivalent surface moieties to molecular volume make these nano-sized materials of interest for the development of synthetic non-viral vectors for therapeutic nucleic acids [36]. Reactive end-groups of dendrimers allow the addition of repetitive units or branching in a controllable manner and versatility by modification of the end groups for multiple copies of various ligands including therapeutics and imaging agents for biomedical applications [38,87].

Synthesis of dendrimer-type molecules was first reported as 'cascade molecules' [88]. Some years later, Tomalia and co-workers pioneered the synthesis of this new class of polymer and refer to them as starburst “polymers” or “dendrimers” [89,90]. Dendrimers containing poly(amidoamine) (PAMAM) as the branching unit have been studied extensively compared to other classes of dendrimers. Dendrimers differ from classical random coil macromolecules due to three distinct characteristics: a central core, layers of repetitive units (termed generations) radically attached to the central core, and the presence of terminal functional groups amenable to conjugation for ligand attachments.

## 7. Synthesis

**Figure 2:**
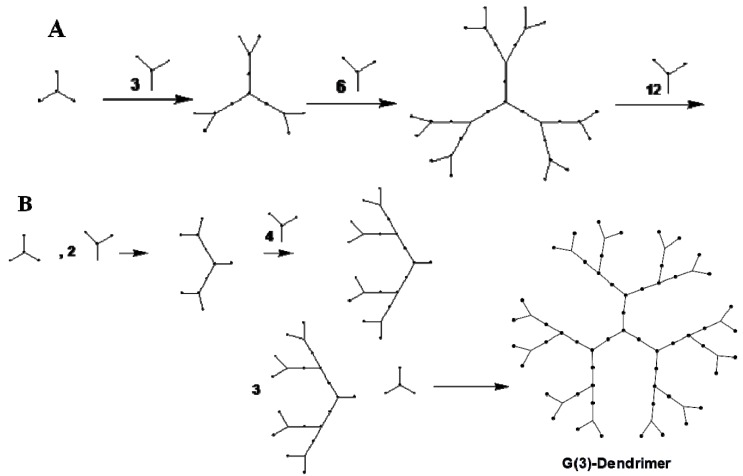
Two common synthetic approaches of dendrimer assembly. Divergent (**A**) amd convergent (**B**) growth method.

Dendrimers can be synthesized by two different approaches: divergent and convergent synthesis (Figure 2). Divergent synthesis involves outward, repeated addition of monomers or branching, starting from a multifunctional core by a series of stepwise polymerization reactions that attach layers of repetitive units/monomers to form dendrimers of various generations [89,91].

**Figure 3:**
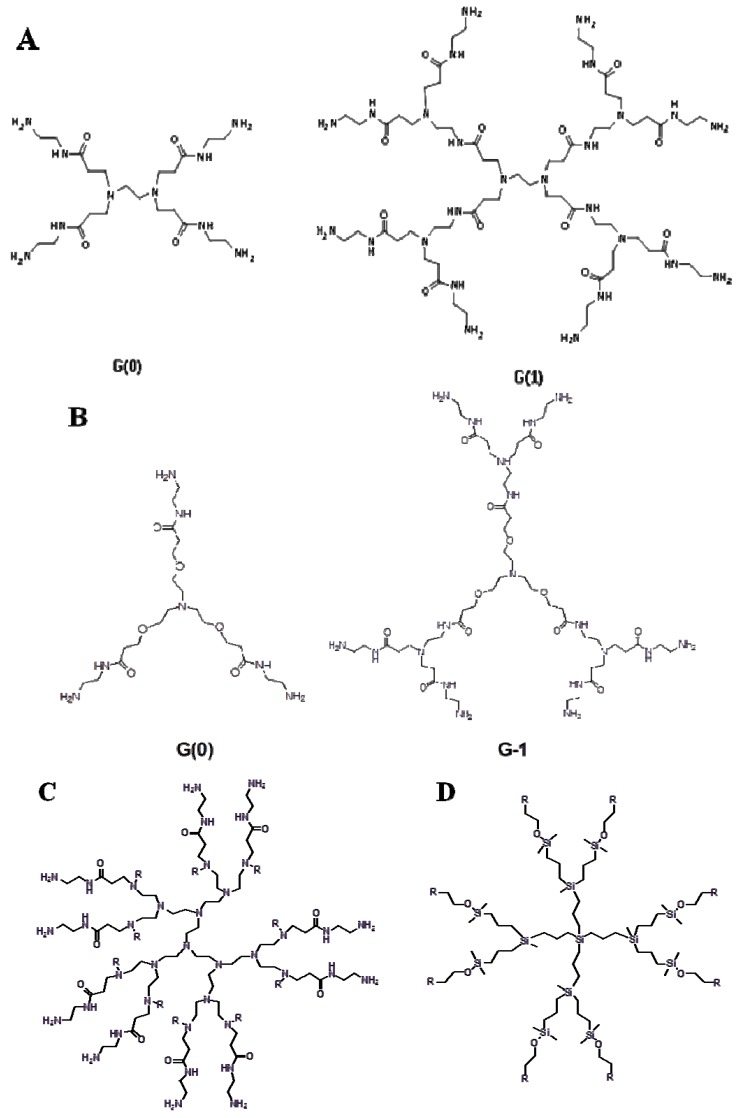
Schematic representation of PAMAM dendrimers with-**A**, ethylenediamine; **B**, triethanolamine; **C**, PEI-PAMAM as the core; **D**, a carbosilane dendrimer of second generation.

The valency of the core determines the starting number of branching points. For example, the ethylenediamine core in poly(amidoamine) dendrimers has four branching points (Figure 3A). The addition of four branchings of a repeating unit (-CH_2_-CH_2_-CONH-CH_2_-CH_2_-N-) generates a PAMAM dendrimer with four primary amine groups on the surface and two tertiary amines in the interior (referred to as generation 0). Addition of repeated branching makes PAMAM dendrimers of G=1, 2, 3, 4, 5 and so on with the number of exterior primary amine groups equal to 8, 16, 32, 64, 128 and so on. A schematic representation of various dendrimers is depicted in Figure 3. The convergent synthetic approach involves inward branching from the dendrimer surface to the inner core by formation of individual dendrons [92]. The dendrons are produced by repeated coupling (depending on the generation) of the branching units which are finally anchored to the core (Figure 2B). Both synthetic procedures pose a challenge for large scale production. Various alternative methods have been proposed [80,93,94,95]. Attempts have been made to reduce the reaction steps involved in the synthesis of dendrimers by the two procedures outlined above [94,95].

Non-symmetrical dendrimers containing both a mannose binding and coumarin fluorescent unit have been prepared by high yield click chemistry of copper(I)-catalyzed azide-alkyne cycloadditions [95]. Maraval *et al*. developed a new straight forward synthesis method of dendrimers using two branched monomers where each generation was obtained in a single quantitative step [94]. PAMAM dendrimers are generally synthesized by the divergent approach. The initiator core is based on ethylene diamine or ammonia (Figure 3A, B). Addition of each new layer increases the molecular weight exponentially; the number or surface-active groups doubles and the diameter increases about 10 Å [37]. PAMAM dendrimers with high generation numbers have a high density of primary amine groups on the surface which make them efficient for binding nucleic acid molecules. Dendrimers possess low polydispersity. An increase in the generation number affects the shape of a dendrimer. Dendrimers of lower generations have a planar or elliptical shape, whereas dendrimers of higher generations typically have a spherical structure with a hydrophobic interior core, useful for the encapsulation of bioactive molecules [37].

Since their discovery, PAMAM dendrimers have gained interest as non-viral nucleic acid delivery vectors due to their high aqueous solubility, high transfection efficiency and minimal cytotoxicity. The positively charged primary amine groups on the surface of these dendrimers allows electrostatic interaction with negatively charged DNA molecules. The PAMAM dendrimer-nucleic acid complex can also be stable over a broad pH range [96].

## 8. Dendrimers as siRNA Delivery Vectors

Dendrimers are among the most widely used cationic polymers for nucleic acid delivery. However, as was said earlier, delivery of siRNA in particular is relatively unexplored. Several different formulations of dendrimer-DNA polyplexes were investigated including PAMAM-DNA, polyethylene glycol-modified PAMAM-DNA, PAMAM-PEG-PAMAM-DNA, PPI-DNA and PEI-DNA [39,97,98,99,100]. The relatively inflexible siRNA experiences a less efficient interaction with cationic polymers compared to DNA, indicating a cationic polymer efficient for plasmid DNA delivery may not be an effective siRNA carrier.

To elucidate the molecular mechanism of PAMAM-siRNA dendriplex self-assembly, Jensen *et al.* studied different generation PMAM dendrimers [101]. G4 and G7 displayed an equal efficiency for dendriplex formation. However, G1 with less charge density lacked the siRNA condensation ability. Reduced average size and increased polydispersity at a higher dendrimer concentration indicated a thermodynamically favorable electrostatic attraction. A spontaneous exothermic binding for G1 and a biphasic, initial exothermic and secondary endothermic binding with siRNA for G4 and G7 was observed for the formation of dendrimer-siRNA aggregates. Flexible G1 and rigid G7 displayed an entropic penalty, making G4 the most suitable for dendriplex formation with a favorable charge density for siRNA binding.

## 9. Dendrimers with Varied Structures for siRNA Delivery

Dendrimers with varied core/branching structures have been utilized for siRNA delivery [20,63,79,102,103]. Polymerized PEG-based dendrimeric core-shell structures including polyglycerolamine (PG-Amine), polyglyceryl pentaethylenehexamine carbamate, PEI-PAMAM and PEI-gluconolactone were synthesized and tested for their efficacy as siRNA delivery vectors. A representative structure of a PEI-PAMAM dendrimer is shown in Figure 3C. The study indicated that these cationic dendrimers exhibited low toxicity, strongly improved the stability of the siRNA and its intracellular trafficking and had both *in vitro* and *in vivo* gene silencing efficacy. These dendritic polymers demonstrated high efficacy in silencing the luciferase gene, ectopically over-expressed in human glioblastoma and murine mammary adenocarcinoma cells. The data demonstrated that the PG-amine exhibited the best ratio of silencing efficacy *vs.* toxicity *in vitro*. Intratumoral and intravenous administration of luciferase targeting siRNA-PG-amine polyplexes to tumor-bearing mice resulted in a significant luciferase gene silencing effect within 24 h of treatment. High luciferase gene silencing (68% and 69%) was accomplished *in vivo* within 24 h of treatment with 2.5 mg/kg of luciferase siRNA in subcutaneously implanted U87-Luc human glioblastoma cells in SKID mice and DA3-mCherry-Luc cells in BALB/C mice with both *i.v.* and *i.t*. dosing respectively.

A family of triazine dendrimers, differing in their core flexibility, generation number, and surface functionality was developed using a divergent synthesis approach and evaluated for its ability to condense and effectively deliver a luciferase targeting siRNA for target-specific knockdown of the reporter gene [63]. The triazine groups were introduced in the periphery, which was linked by specific diamine groups. The triazine dendrimers, which was the most effective DNA delivery vector, was not able to mediate gene silencing, whereas the moderately effective gene delivery vector delivered significant amount of siRNA [102,104]. A molecular modeling and *in vivo* imaging study were performed to identify a useful flexible triazine dendrimer for siRNA delivery. In comparison with PEI, 25 KDa, flexible G2-4 triazine dendrimers formed thermodynamically more stable complexes with siRNA and demonstrated less toxicity. Triazine dendrimer-based siRNA delivery systems were more efficiently charge-neutralized than PEI complexes, which reduced agglomeration. The hydrodynamic diameters ranged from 72.0 to 153.5 nm for dendriplexes compared to 312.8 to 480.0 nm for PEI-siRNA complexes. All dendriplexes were efficiently endocytosed and demonstrated significantly higher luciferase knockdown. However, dendriplexes of higher generation were captured by reticuloendothelial system due to their increased surface charge.

Another new class, carbosilane dendrimer, has been utilized for siRNA delivery (Figure 3D) [103,105]. Amino terminated carbosilane dendrimers were developed to protect and transport siRNA to HIV-infected lymphocytes [103]. The dendrimers were able to effectively bound siRNA via electrostatic interactions, and the dendrimer-bound siRNA was resistant to degradation by RNAse. The dendriplexes with a N/P ratio of 2 displayed the highest transfection efficiency with no cytotoxicity in hard-to-transfect HIV-infected peripheral blood mononuclear cells. The dendrimer-siRNA complex down-regulated GAPDH expression and reduced HIV-replication in the tested lymphocytic cell lines. Carbosilane dendrimers were also utilized to deliver siRNA to postmitotic neurons to study the function of hypoxia-inducible factor-1 alpha (HIF1-alpha) during chemical hypoxia-mediated neurotoxicity [105]. Carbosilane dendrimers were as effective as viral vectors in terms of their siRNA delivery and transfection efficiency. Carbosilane dendrimers with sixteen positive charges/molecule caused strong repression of various interleukins in macrophages involved in autoimmune diseases suggesting a potential pharmacological application of this dendrimer [106].

Polypropyleneimine (PPI) dendrimers appear to be an attractive non-viral vector for siRNA delivery [68]. A PPI dendrimer-siRNA nanoparticle was formulated using a layer-by-layer surface modification strategy, where the nanoparticle was caged with a dithiol containing cross linker molecule followed by coating with a poly(ethylene glycol) polymer to provide the stability to the nanoparticles needed to withstand the neutralizing environment in the blood stream. Specific cancer targeting of the nanoparticles was achieved by conjugating a cancer-homing peptide, luteinizing hormone-releasing hormone (LHRH) peptide to the distal end of the PEG chain. The disulfide bonds of the coated nanoparticles get reduced after cell uptake and the nucleic acid is released in to the cytoplasm [107]. The coating with a dithiol linkage on the dendriplex provides stability and leads to less agglomeration of the nanoparticles during their circulation [108]. However, this could also cause decreased transfection efficiency due to over-stabilization of the product and require utilization of less stable disulfide linkages [109]. The surface functionalization with a thiol linkage was useful in stabilizing the nanoparticles in terms of dissociation in presence of competing polyanions. The modified nanoparticles were stable in human serum for at least 48 h. PEGylation imparted stability against aggregation by decreasing the particle-particle and particle-protein interactions. The functionalization of the nanoparticle surface with LHRH peptide for cancer targeting should be an effective strategy. LHRH receptors are over-expressed in several types of cancer including ovarian, prostate, lung, breast, and colon cancer with undetectable expression in healthy visceral organs [110]. The results obtained from this *in vivo* biodistribution study indicated a high degree of nanoparticle accumulation in tumor compared to other organs.

Hayashi *et al.* used a lactose group-modified, α-cyclodextrin conjugated G3-dendrimer (Lac-α-CDE) for the delivery of hepatocyte-specific siRNA carriers for the treatment of transthyretin-related familial amyloidotic polyneuropathy [111,112]. Lac-α-CDE was condensed with a siRNA that targets transthyretin (TTR) gene expression. The dendrimer-siRNA complex had a potent RNAi effect against the TTR gene expression, efficient endosomal escape and delivery of the siRNA complex to the cytoplasm with negligible cytotoxicity. After intravenous administration, The Lac-α-CDE/siRNA complex demonstrated a potent *in vivo* gene silencing effect.

A flexible PAMAM dendrimer with triethanolamine (TEA) as the core, in which the branching units start at a distance of 10 successive bonds from the center amine has also been studied for siRNA delivery and gene silencing [29,113,114,115]. A TEA core offers flexibility compared to the commercially available PAMAM with an ammonia or ethylenediamine core, in which the branching starts at the central amine of the core. Higher generation dendrimers of this family can efficiently deliver siRNA and induce gene silencing. Dendrimers with amine end groups almost completely retarded siRNA in the agarose gel electrophoresis at N/P ratios above 2.5. However, dendrimers with terminal ester groups demonstrated no gel retardation, indicating no condensation with siRNA. TEA core PAMAM dendrimers efficiently delivered siRNA and inhibited the catalytic activity of *Candida* ribozymes [115]. A TEA core PAMAM dendrimer delivered HSP27-targeted siRNA into prostate cancer cells [113]. This dendrimer, which protects siRNA from enzymatic degradation, enhanced cellular uptake of siRNA. The siRNA also exhibited potent and specific gene silencing of heat shock protein 27, which is an attractive therapeutic target in castration-resistant prostate cancer.

## 10. Surface Modification for Improved Efficacy and Multifunctionality

Surface functionalization of nanocarriers with a wide variety of polymers and targeting ligands is a promising approach to achieve specific functions [116]. Surface modification of nanocarriers with a biocompatible and hydrophilic polymer, poly(ethylene glycol) (PEG), has found wide application as pegylation shields the nanocarrier from exposure to enzymes or opsonizing proteins in the systemic circulation [74,117,118,119,120,121]. Evading capture by the reticuloendothelial system provides prolonged systemic circulation, which is particularly essential for nanocarriers that eventually accumulate in an infarcted region or tumor via the enhanced permeability and retention (EPR) effect [74,122,123]. PEG-conjugation decreased the cytotoxicity of cationic polymers for nucleic acid delivery such as PLL and PEI by reducing or partially shielding the positive charge on the surface of these polycations [74,118,119,121,124]. PAMAM dendrimers of higher generations such as G4 and G5 are highly efficient as nucleic acid delivery vectors, However their high cytotoxicity, liver toxicity and hemolysis limit their *in vivo* application [91,125,126].

PEG-modified PAMAM dendrimers have demonstrated low toxicity and mediated efficient drug and gene delivery [127,128,129]. The amount of PEG on the surface affects its transfection efficacy and cytotoxicity. The G(5) PAMAM, conjugated to 10% PEG-3.4 KDa had a 20-fold increase in the efficacy of *in vitro* gene transfection compared to the unconjugated G(5) PAMAM dendrimer [130]. Qi *et al.* demonstrated that 8 mol % PEG-conjugated G5 and G6 dendrimers were the most efficient at gene silencing, when compared to three pegylated systems of G5 and G6 PAMAM dendrimers at a 4, 8, or 15% molar ratio of PEG on the surface [127].

PAMAM dendrimers have been surface functionalized for selective siRNA delivery. G(5)-PAMAM dendrimers were conjugated to cell penetrating TAT peptide for the purpose of intracellular delivery [131]. *MDR1* gene silencing siRNA-dendrimer polyplexes weakly inhibited the gene expression. In this study, conjugation with TAT-peptide did not improve the delivery efficiency of the G(5)-PAMAM dendrimer. However, TAT-peptide functionalized dendrimer-oligonucleotide complexes were moderately effective for delivery of antisense compared to plain dendrimer. The effect of acetylation of primary amines of G5 on dendriplex formation was also studied [132]. The results indicated that acetylation reduced the cytotoxicity and that approximately 20% of the primary amines of G5-PAMAM could be modified while maintaining the siRNA delivery efficiency seen with unmodified PAMAM. A higher degree of amine neutralization reduced cellular delivery, caused entrapment in the endosome due to the reduction in buffering capacity and reduced gene silencing efficiency.

A novel approach to reducing the cytotoxicity of the dendrimers was outlined by the Minko group [133,134,135,136]. They evaluated the internally cationic, surface neutral dendrimers as nanocarriers for the targeted delivery of siRNA. An internally quaternized and surface acetylated PAMAM-G4 dendrimer was synthesized. The neutral surface of these dendrimers elicited low cytotoxicity compared to parent dendrimer with amino groups on the surface. Interaction of the siRNA with the cationic charge inside the dendrimer resulted in the formation of a compact nanoparticle, that potentially protects siRNA from environmental degradation. The shape of internally quaternized, surface neutral PAMAM/siRNA polyplexes at a charge N/P ratio of 3 was spherical, whereas condensation of siRNA with PAMAM-NH_2_ at the same N/P ratio resulted in the formation of ribbon-like nanofibers [134]. PAMAM-OH dendrimers were internally positively charged by the reaction of tertiary amines with methyl iodide, and this charge was utilized for siRNA condensation. Internal condensation of siRNA played a significant role in controlling the morphology of the complexes by encapsulating the siRNA in the core. Quaternized G4-PAMAM-OH dendrimers (QPAMAM-OH) were functionalized with a synthetic analog of the natural LHRH peptide for active cancer targeting [136]. LHRH peptide conjugated QPAMAM-OH-LHRH successfully targeted cancer cells and facilitated cellular uptake of dendrimer-siRNA complex via interaction with over-expressed LHRH receptors through receptor-mediated endocytosis and accumulated in the tumors, with minimal invasion of the healthy tissues to potentially limit adverse side-effects.

**Figure 4:**
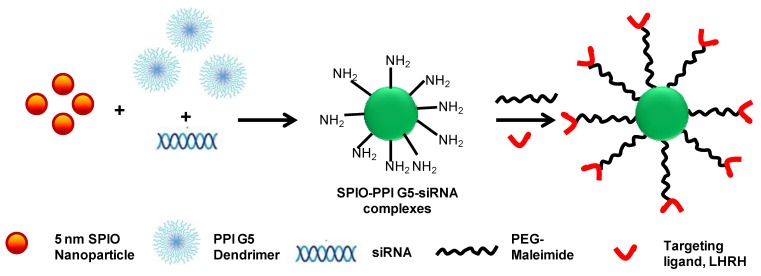
Schematic representation of the development of a cancer cell-targeted dendrimer-siRNA-SPION complex.

Functionalized inorganic nanomaterials including gold, iron oxide nanoparticles, quantum dots and carbon nanotubes provide a promising platform for siRNA delivery since these materials produce a high molecular weight polymer structure with functional groups on the surface [137,138]. The inorganic core functions as a space-filling material that presents surface-associated active functional groups. In a recent study, gold nanoparticles were functionalized with biodegradable glutamic acid scaffolds and cationic triethylenetetramine terminated dendron ligands for effective electrostatic interaction with siRNA [137]. In another study, poly(propyleneimine) generation 5 dendrimers (PPI G5) were cooperatively complexed with super paramagnetic iron oxide nanoparticles (SPION) and siRNA to develop a complex tumor-targeted drug delivery system for simultaneous delivery of siRNA with this MRI contrast agent specifically to cancer cells [138]. A schematic representation of the concept is shown in Figure 4. The dendrimer-siRNA-SPION particles were stabilized with PEG, the distal end of which was coupled with the tumor homing peptide, LHRH. This multi-functional system delivered siRNA specifically to the cancer cells and allowed monitoring of the therapeutic outcome using simultaneous imaging techniques.

In our recent study, PAMAM-G4 dendrimer was lipid-modified for siRNA-drug co-delivery [139]. We synthesized a triblock co-polymeric system by conjugating G(4)-PAMAM dendrimer with poly(ethyleneglycol)-1,2-dioleoyl-sn-glycero-3-phosphoethanolamine (PEG-PE). The lipid block in the polymer provided optimum hydrophobicity and compatible cellular interaction for enhanced cell penetration. A mixed micellar system was developed using G(4)-D-PEG-PE and PEG-PE polymer at a 1:1 molar ratio. The lipid-modified, PEGylated dendrimer, G(4)-D-PEG-PE and the mixed micellar system form stable complexes with siRNA, showed excellent serum stability and significantly higher cellular uptake of siRNA that resulted in better targeted green fluorescence protein (GFP) downregulation compared to G(4)-PAMAM dendrimer. The core of the mixed micellar system loaded a chemotherapeutic drug, doxorubicin, efficiently, while the PEG-PE anchored G(4)-D condensed siRNA. The modified dendrimer demonstrated higher efficiency for siRNA delivery compared to G(4)-D and the mixed micellar system. The mixed micellar system appeared to be a promising career for siRNA/drug co-delivery.

**Figure 5:**
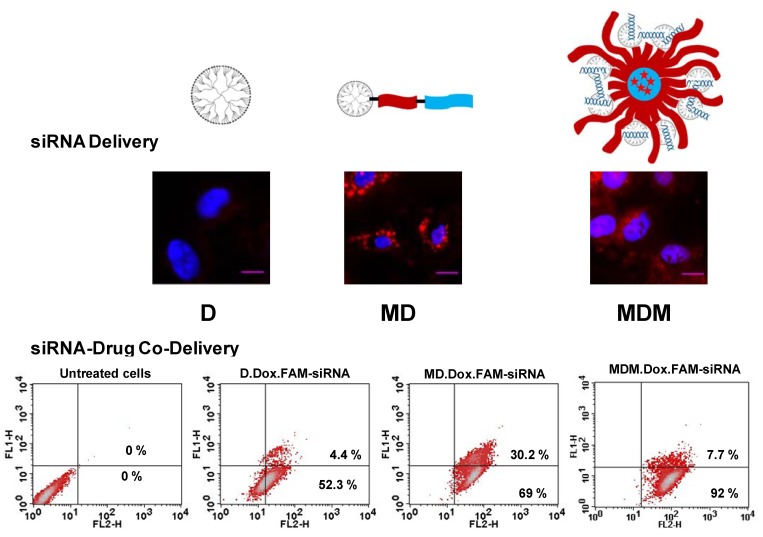
Lipid modification of G(4)-PAMAM dendrimers for enhanced siRNA delivery and siRNA-drug co-delivery. Green fluorescence of FAM-labeled siRNA and red fluorescence of Doxorubicin were analyzed by flow cytometry to assess siRNA/drug co-delivery.

Figure 5 showed confocal microscopy images of the dendrimer (D), lipid-modified dendrimer (MD), and mixed micellar system (MDM)-dosed cells, used to assess the intracellular siRNA delivery efficiency.

The flow cytometry data representing the efficiency of the polymer for siRNA-drug co-delivery are also shown. This study clearly demonstrated that lipid modification enhances the cellular association of the nanocarrier without compromising the siRNA condensation ability of the dendrimers. Moreover, a mixed micellar system is advantageous for its ability to simultaneously carry and effectively deliver both drug and siRNA.

## 11. Conclusions

In summary, dendrimer-mediated delivery of therapeutics including siRNA clearly should be considered a promising approach. Significant research is being carried out to develop dendrimer-based nanomedicine for the delivery of drugs and nucleic acids. The unique properties of this polymeric system, including ease of surface functionalization, enables engineering of truly multifunctional nanodevices for drug delivery applications. However, further addressing of the current limitations including non-specific cytotoxicity associated with higher generation-dendrimers, release kinetics of the associated bio-actives and rapid clearance issues would open up new perspectives for dendrimers as therapeutic nanoparticles.
